# Eccrine sweat reveals normalization of artificial sweetener levels in children with overweight after nutritional education

**DOI:** 10.1016/j.isci.2025.113736

**Published:** 2025-10-11

**Authors:** Michael Wolf, Daniel Wasinger, Viktoria Donhauser, Paula Moliterno, Bernhard K. Keppler, Christopher Gerner, Kurt Widhalm, Samuel M. Meier-Menches

**Affiliations:** 1 Institute of Analytical Chemistry, University of Vienna, Vienna, Austria; 2 Doctoral School of Chemistry, University of Vienna, Vienna, Austria; 3 Austrian Academic Institute of Clinical Nutrition, Vienna, Austria; 4 Division of Clinical Nutrition and Prevention, Department of Pediatrics, Medical University of Vienna, Vienna, Austria; 5 Institute of Inorganic Chemistry, University of Vienna, Vienna, Austria; 6 Joint Metabolome Facility, Medical University of Vienna and University of Vienna, Vienna, Austria

**Keywords:** Human metabolism, Metabolomics, Nutrition education

## Abstract

Determining metabolic parameters associated with childhood obesity is challenging due to the invasive nature of most sampling methods. Using a non-invasive eccrine sweat collection, 134 metabolomes were obtained from 54 children, of which 20% had overweight, including obesity. This cohort is part of the preventive EDDY program aiming to promote healthy lifestyles in school-aged children from Vienna, Austria. The abundance of the artificial sweetener cyclamate in eccrine sweat correlated with body mass index in the overweight group at baseline. Cyclamate was also found to be elevated in the overweight group at baseline compared to the normal weight group, but normalized after the intervention, indicating a positive impact of the EDDY program. Furthermore, dynamic changes in metabolites originating from the gut microbiome were observed. Sweat metabotyping thus represents a valuable approach to accessing metabolic parameters in children in the context of overweight and obesity and promises broad applicability in pediatric research.

## Highlights

• Sweat metabolomes of children are reported based on non-invasive sample collection
• Normal weight and overweight children display differences in sweat metabolomes
• The artificial sweetener cyclamate was elevated in the overweight group at baseline
• Cyclamate abundance in the overweight group normalized after the intervention

## Graphical abstract

**Figure:**
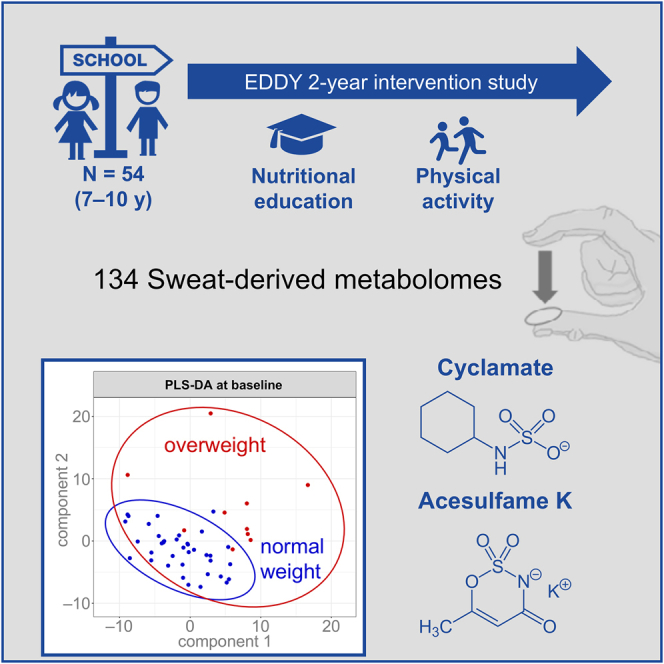

## Introduction

Childhood overweight and obesity are characterized by excessive fat deposits in the body and are enormous public health concerns. Overweight in children of the same sex and age is defined according to national reference data by a body mass index (BMI) at or above the 90^th^ percentile, while obesity is defined by a BMI at or above the 95^th^ percentile.^1^ From 1975 to 2016, the worldwide prevalence of childhood obesity increased substantially.^2^ According to the World Health Organization 24.4% and 29.5% of girls and boys, respectively, have overweight, including obesity.^3^ Although there seems to be a stabilization of excessive body weight trends in Western countries,^4^ it is expected that the worldwide prevalence doubles until 2035 www.worldobesityday.org.^5^ Importantly, childhood obesity is associated with a higher likelihood of lifelong excess body weight.^6^ Overweight and obesity are risk factors for cardiovascular diseases and diabetes mellitus type 2, and negatively influence longevity.^7^

Childhood overweight has multifactorial causes that are linked to genetics, individual choices, lifestyle, education, as well as cultural and socio-economic contexts.^7^ It is therefore challenging to devise effective preventive strategies, which are ideally based on multi-component approaches.^8^^,^^9^ The need for interventions with respect to physical activity was already proposed.^10^ Meta analyses revealed a positive impact on overweight by dietary intervention and physical activity,^11^ although the beneficial effect of the former did not lead to an objective improvement in some cases.^12^ Interestingly, it was also found that suitably controlled community initiatives and frameworks could build awareness for complex interventions to address childhood overweight.^13^ The EDDY program (acronym for “**E**ffect of sports and **D**iet training to prevent obesity and secondary **D**iseases and to influence **Y**oung children’s lifestyle”) aims to reduce overweight and obesity in children^14^^,^^15^^,^^16^ in Austria, a country for which the World Obesity Federation assigned a high childhood obesity risk score.^17^ The EDDY study developed a dual intervention based on dietary education and increased physical exercise in young children (mainly 7–10 years) and was shown to have beneficial effects by reducing the overweight percentage.^14^^,^^15^^,^^18^

In practice, pediatric studies about childhood overweight and obesity typically assess anthropometric parameters, BMI or body composition, or dietary habits based on questionnaires. Accessing molecular data of children is challenging because most sampling techniques are invasive, drawing blood. Sweat is increasingly recognized as a diagnostic biofluid^19^ and can be obtained by non-invasive sampling. In fact, some metabolites were successfully detected in sweat-derived fingerprints and were used to image latent fingerprints.^20^ More recently, it was realized that sweat of a fingerprint could be used as a drug screening method,^21^^,^^22^ and for forensic purposes.^23^ It was further found that sweat would contain low amounts of extracellular vesicles (EVs) that could be enriched and used for biomarker identification.^24^^,^^25^ These EVs were shown to contain distinct metabolites depending on exercise.^26^

Sweat collection is potentially suitable for sampling children to access metabolic parameters and thus, molecular effects of the EDDY intervention. In contrast to microfluidic systems that assess a limited number of parameters,^27^^,^^28^ or clinical-grade patches,^29^ we have established a simple technique based on insensible eccrine sweat collection from fingertips combined with a mass spectrometry-based analysis platform to discover hundreds of exogenous and endogenous metabolites in eccrine sweat,^30^ which can be used to monitor molecular lifestyle parameters.^31^ This approach was analytically validated, does not require trained personnel and is based on collecting eccrine sweat without sweat induction.^30^ Eccrine sweat glands are responsible for thermoregulation^32^ and can be associated with drug excretion,^33^ a phenomenon that was also evidenced in our approach.^34^ It is known that eccrine sweat is derived from the interstitial fluid,^32^^,^^35^ which might explain its unexpected complex composition.

In this feasibility study, we address the sample collection challenge in pediatric research and show that eccrine sweat can be collected efficiently from children and with good compliance. Sweat metabolomics data provides an unprecedented level of molecular information about endogenous metabolites and lifestyle parameters in children. With this approach, dynamic changes in metabolism and lifestyle parameters, especially non-caloric artificial sweeteners, were successfully detected in the eccrine sweat of a sample of school-aged children with overweight and obesity and capture the effect of an extended two-year dual intervention of the EDDY program in Vienna, Austria.

## Results

### Sweat collection from the fingertips enables the determination of molecular parameters in children

**Figure 1:**
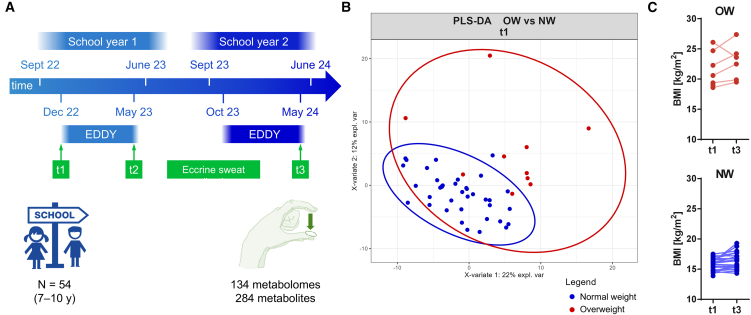
Eccrine sweat was collected from School children (7–10 years) over the course of a two-year dual intervention, based on the EDDY program (A) Timeline of the EDDY intervention. The children underwent dietary education and physical exercise in the frame of the school curriculum of 3^rd^ and 4^th^ grades. Eccrine sweat was collected at baseline (t1), at the end of 3^rd^ grade (t2) and after completion of the intervention at the end of 4^th^ grade (t3). Body mass index (BMI) was assessed at baseline (t1) and after completion of the intervention (t3). A total of 284 metabolites were annotated in 134 acquired sweat metabolomes of 54 children. (B) The 284 metabolites were used for partial least-squares discriminant analysis (PLS-DA) of all available participants at baseline (t1) according to normal weight (NW, *n* = 37, blue) and overweight (OW, *n* = 10, red) children. (C) Assessments of BMI at baseline (t1) and after completion of the intervention (t3) according to children with normal weight (NW, *n* = 22, blue) and overweight (OW, *n* = 7, red) for whom these data was available. The changes from t1 to t3 were not statistically significant using an unpaired *t* test with Welch’s correction, *p* value (NW, *n* = 22) = 0.07, *p* value (OW, *n* = 7) = 0.44.

The EDDY intervention represents a prospective intervention, involving physical activity and dietary education and was extended to two years in a non-representative sample of children of 3^rd^ and 4^th^ grade in Vienna, Austria. Eccrine sweat of the children was collected at three time points, including baseline (t1), after one school year (t2) and after the second school year (t3) at completion of the intervention. BMI was assessed at baseline (t1) and after completion of the intervention (t3). Sweat collection was performed in School and was well accepted among the children. A total of 134 sweat samples were collected of 54 children of which 20% had overweight, including obesity (Table 1). We obtained complete sample sets of 31 children, representing a response rate of 57%. Samples were collected by firmly pressing the thumb and index+middle fingers of the non-dominant hand on a prewetted substrate (Figure 1A). The children in this study used their index and middle fingers together to cover more surface area for sampling. Metabolomics data were acquired similarly to a previous study.^30^ An improved evaluation method based on empirical Bayes normalization was used to account for batch effects. Eccrine sweat of the children revealed 284 annotated metabolites in positive and negative ion modes (>60% confidence, Supplementary Document S1). Partial least-squares discriminant analysis (PLS-DA) of the sweat metabolomes of the available normal weight (*n* = 37) and overweight (*n* = 10) children at baseline (t1) revealed a partial separation of the two groups (Figure 1B). As expected for the otherwise healthy children, we found only a minor metabolic impact of overweight on the metabolome in eccrine sweat. Importantly, among the metabolites that contribute most to the separation between normal weight and overweight children, we found the non-caloric artificial sweetener cyclamate, which is used in many beverages and foods as a source of sweetness without calories. The most common include acesulfame K, aspartame, cyclamate, saccharin, sucralose, and stevia.^36^ The BMI of the children in the normal weight and overweight groups did not change in a statistically significant manner between baseline and after completion of the 2-year intervention, suggesting a minor impact of the intervention on actual bodyweight (Figure 1C).

**Table 1:** Description of the study cohort in the intervention School of the EDDY program. Arithmetic means are given with standard deviations (n = number of participants of each group). BMI = body mass index

	Total Cohort (%)	Complete Sample Sets		
		Total (%)	Female (%)	Male (%)
N° participants	54	31	12	19
N° normal weight	43 (80%)	23 (74%)	9 (75%)	14 (74%)
N° overweight	11 (20%)	8 (26%)	3 (25%)	5 (26%)
BMI [kg m^−2^]	17.3 ± 3.3	17.6 ± 3.9	17.7 ± 3.9	17.5 ± 4.0
Age [years]	7.9 ± 0.7	7.7 ± 0.6	7.7 ± 0.5	7.7 ± 0.7

### Eccrine sweat reveals dynamic changes in levels of artificial sweeteners over the course of the EDDY intervention

**Figure 2:**
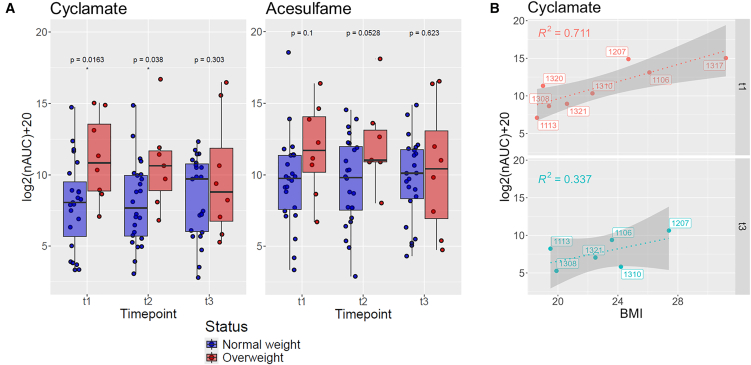
Abundance of artificial sweeteners cyclamate and acesulfame in eccrine sweat of children according to weight status (A) Time-dependence of the abundance of the artificial sweeteners in eccrine sweat. Intensities are given as log2-transformed normalized area under the curves [log2(nAUC)+20]. Statistical significance was calculated by fitting linear models using the limma package in R (n(NW) = 23, n(OW) = 8), ∗ = *p* value <0.05. (B) Correlation of cyclamate abundance in eccrine sweat with BMI for the overweight group according to baseline (t1, *n* = 8) and after completion of the intervention (t3, *n* = 6). Labels correspond to individuals and colors to time points of sampling (R^2^(all data points) = 0.15).

The successful separation of overweight from normal weight phenotypes based on the eccrine sweat metabolome in children at baseline using PLS-DA motivated the evaluation of the impact of the EDDY program on sweat phenotypes over the 2-year intervention. Indeed, cyclamate was found to be elevated in overweight children at baseline measurements (Figure 2A). Acesulfame represents another widely used artificial sweetener and was also found at elevated levels at baseline in overweight children, albeit not as pronounced as cyclamate (Figure 2A). The abundance of cyclamate in the overweight group gradually normalized during the EDDY intervention compared to the normal weight group. This suggested a cumulative effect of the intervention over two years and may reflect a reduction in the excess intake of products containing artificial sweeteners. Part of the nutritional education intervention was to increase children’s knowledge about the difference between ingredients in pre-packaged foods and beverages and natural ones. In addition, great emphasis was placed on teaching about limiting the consumption of sugary drinks (regular and diet drinks) and to recommend water as a valuable substitute. Moreover, a correlation between cyclamate abundance in eccrine sweat and BMI was observed in the overweight group at baseline (t1), suggesting that this may indeed reflect lifestyle behavior (R^2^ = 0.71, Figure 2B). Children with excessive body weight may not only register higher caloric intakes than those with normal weight but also consume a broader range of products containing single or mixed non-caloric sweeteners. This association was not observed after completion of the intervention at t3 (R^2^ = 0.34), when including the normal weight children at the same time point (R^2^ = 0.27) or all data points of the overweight group (R^2^ = 0.15).

### Dynamic changes in endogenous metabolites are revealed by sweat metabotyping over the course of the EDDY intervention

**Figure 3:**
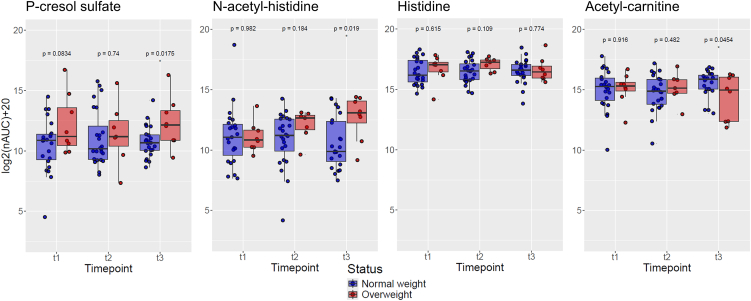
Time-dependence of endogenous gut microbiome-related metabolites in eccrine sweat of children according to weight status Intensities are given as log2-transformed normalized area under the curves [log2(nAUC)+20]. P-cresol sulfate is also known as p-cresyl sulfate. Statistical significance was calculated by fitting linear models using the limma package in R (n(NW) = 23, n(OW) = 8), ∗ = *p* value <0.05.

Next to exogenous, lifestyle-associated metabolites, eccrine sweat also revealed dynamic changes of endogenous metabolites, particularly microbiome-related metabolites, over the course of the EDDY intervention according to weight status (Figure 3). For example, p-cresol sulfate is a uremic toxin^37^ and was previously associated with insulin resistance in conjunction with chronic kidney disease in mice.^38^ This metabolite was found at higher abundance in the overweight group. In an animal model, N-acetylated amino acids were found to be largely derived from the gut microbiome.^39^ N-acetyl histidine was found to gradually increase in the overweight group over the course of the study, while histidine did not show such a trend. Finally, carnitines are known to be metabolized by the gut microbiota.^40^ Acetyl-carnitine was found at reduced abundance in the overweight group after completion of the study. Overall, because of the strong connection of the gut microbiome and human metabolism, sweat metabotyping may be used to infer systemic effects caused by changes in dietary habits and subsequent changes in the composition of the gut microbiome. Again, the 2-year intervention seemed to manifest a more pronounced effect compared to a 1-year intervention.

## Discussion

This study reports a sweat metabolome dataset from children by sweat metabotyping, the assessment of metabolic markers from eccrine sweat from the fingertips. This approach revealed relevant molecular information with respect to lifestyle parameters and endogenous metabolites in the context of childhood overweight and obesity. Eccrine sweat collection is non-invasive and does not require trained personnel, which facilitates implementing this approach in pediatric studies and improves compliance in comparison to invasive sampling techniques. This was reflected in a 57% response rate across three sampling points over this 2-year study. Here, response rate refers to the percentage of children that completed all three time points. A total of 284 metabolites were annotated in 134 eccrine sweat metabolomes of 54 children, aged 7–10 years, of which 20% had overweight. The sweat metabolome revealed a minor contrast between normal weight and overweight children according to partial least square discriminant analysis. It was found that the artificial sweetener cyclamate was among the main contributors to the separation between normal weight and overweight children in our dataset. As ingested cyclamate in pregnant women was found to distribute even across the placenta,^41^ there is strong basis to expect that the cyclamate detected in eccrine sweat of children in this study was actually ingested. Non-caloric sweeteners are food additives widely used in beverages, pre-packaged foods, and other products to provide sweetness without additional calories. The World Health Organization has recommended to limit the intake of free sugars as a strategy to address high obesity rates among children worldwide.^36^ Consequently, the industry has reformulated many food products, incorporating non-caloric sweeteners as partial or total sucrose replacement. Thus, children are easily exposed to these products daily, whether their caregivers pursue a weight control objective, or not.^42^ In fact, artificial sweeteners potentially represent unhealthy lifestyle parameters^43^ that are critically discussed in the context of childhood obesity,^44^^,^^45^ and also adult obesity.^46^^,^^47^ In this study, the artificial sweetener cyclamate was detected at elevated abundance in the overweight group compared to normal weight group at baseline. It is believed that nutritional education builds awareness about healthy foods in children, which would ideally lead to a behavioral change to consume less foods and beverages containing the reported artificial sweeteners. Furthermore, a healthier dietary habit would translate to adaptation in the gut microbiome-related metabolome. Indeed, cyclamate abundance levels normalized in the overweight group after the 2-year EDDY program, suggesting a cumulative effect of the intervention. A similar trend was observed for acesulfame. Based on questionnaires, the EDDY program was already suggested to reduce the consumption of junk food, sweets and snacks after a 1-year intervention.^16^ The present study corroborates this earlier finding with molecular evidence. The correlation between cyclamate abundance in eccrine sweat from the fingertip and BMI at baseline in the overweight group was noteworthy, displaying an R^2^ = 0.71, which contrasted the absence of a similar correlation after completion of the intervention (R^2^ = 0.34), in the normal weight group (R^2^ = 0.27) or in all data points of the overweight group, yielding an R^2^ = 0.15. At the same time, the BMI at baseline and after completion of the study did not change in a statistically significant manner in the respective groups. This indicated an increased consumption of cyclamate-containing beverages and products in the overweight group at baseline and a gradual decrease of excess intake over the course of the intervention. The gradual normalization of the abundance of the artificial sweetener cyclamate in the overweight group may therefore represent a consequence of the nutritional education in children. Consequently, this intervention did not lead to a reduction of BMI in the overweight children, but molecular parameters seem to indicate a shift in dietary habits toward reduced consumption of beverages containing artificial sweeteners. It is tempting to speculate that the educational program led at least to temporal adaptations in dietary habits in overweight children, consuming less food products and beverages containing artificial sweeteners.

Finally, metabolites related to the gut microbiome also featured dynamic adaptations over the course of the intervention and underscored the cumulative effect of the 2-year intervention. The complex interplay between changes in dietary habits and consequential changes in the gut microbiome could be responsible for the observed time-dependent changes, but their meaning remains rather speculative. For example, p-cresol sulfate was found elevated in the overweight group consistently throughout the intervention, which might be related to increased protein intake with respect to other macronutrients, carbohydrates. The challenge of inferring meaning in finger sweat data are underscored by our recent report that temporal dynamics of metabolites in the interstitium-derived eccrine sweat can be rather different compared to the dynamics of the same metabolites in blood plasma, possibly due to compartmentalization effects.^34^ In summary, these results show that sweat metabotyping provides access to metabolic parameters in children in a non-invasive manner. As the metabolome of eccrine sweat is being expanded to numerous metabolic markers and lifestyle parameters, this approach promises broad applicability in pediatric studies in cross-sectional studies or longitudinal intervention settings.

### Limitations of the study

Metabolomics of eccrine sweat, termed sweat metabotyping is an emerging technology. It involves a non-invasive sample collection and facilitates human biomonitoring in children. Eccrine sweat originates from the interstitial fluid and the eccrine sweat metabolome is currently expanding considerably due to technological progress.^19^ Despite the promising results obtained with the acquired 134 sweat metabolomes in this feasibility study, the physiology of eccrine sweat glands and the passage of metabolites from blood into eccrine sweat is not yet fully understood. Naturally, non-invasive sample collection of sweat from the fingertips is susceptible to surface contaminations by potential exposure of the skin to food, bacteria, dirt and other origin. This phenomenon is similarly known for hair analysis,^48^ and was addressed by sample collection early morning after washing the hands with lukewarm tap water. Moreover, sweat collection in Schools, as performed in this study, may cause substantial variation, impacting on data interpretation. The conclusions might benefit from an increased power with respect to the number of participants which is planned for future studies. Despite these limitations, this promising approach is unique with regard to collecting metabolic parameters from children in a non-invasive manner and addresses one of the main challenges in pediatric research. Further studies will be conducted to improve the precision and power of this approach, including the elucidation of suitable normalization strategies to improve data quality.

## Resource availability

### Lead contact

Further information and requests for resources should be directed to and will be fulfilled by the lead contact, Samuel M. Meier-Menches (samuel.meier-menches@univie.ac.at).

### Materials availability

This study did not generate new unique reagents.

### Data and code availability

• All data reported in this paper are available from the lead contact upon request.
• This paper does not report original codes.
• Any additional information required to reanalyze the data reported in this paper is available from the lead contact upon request. Raw data can be publicly accessed in the MassIVE data repository under dataset identifier MSV000098910 (https://doi.org/10.25345/C55Q4S029).

## Acknowledgments

The authors acknowledge funding by the 10.13039/501100013699Austrian Federal Ministry of Education, Science and Research. The authors are grateful to the Mass Spectrometry Centre (Faculty of Chemistry, University of Vienna) and the Joint Metabolome Facility (University of Vienna and Medical University of Vienna) for access to the instruments. Both facilities are members of the Vienna Life Science Instruments Consortium (VLSI).

## Author contributions

Conceptualization: K.W., B.K.K., S.M.M.-M., and C.G.; methodology: M.W., D.W., P.M., V.D., S.M.M.-M., and C.G.; investigation: M.W., D.W., P.M., and V.D.; visualization: D.W.; funding acquisition: K.W.; project administration: K.W. and S.M.M.-M.; supervision: K.W.; B.K.K., S.M.M.-M., and C.G.; writing – original draft: M.W., D.W., C.G., K.W., and S.M.M.-M.; writing – review & editing: M.W., D.W., P.M., C.G., K.W., B.K.K., and S.M.M.-M.

## Declaration of interests

Authors declare that they have no competing interests.

## Declaration of generative AI and AI-assisted technologies in the writing process

During the preparation of this work, the authors used deepl.com to improve English style. After using this tool or service, the authors reviewed and edited the content as needed and take full responsibility for the content of the publication.

## STAR★Methods

### Key resources table

**Table:** 

REAGENT or RESOURCE	SOURCE	IDENTIFIER
**Biological samples**		

Eccrine sweat	Fingertip of human	–

**Chemicals, peptides, and recombinant proteins**		

H2O (LC-MS grade)	VWR Chemicals	Cat# 83645.320
MeOH (LC-MS grade)	Honeywell	Cat# 34966-2.5L
Formic acid (LC-MS grade)	VWR Chemicals	Cat# 84865.180
Caffeine-d9 (99 atom %D)	Merck (Sigma-Aldrich)	Cat# 725625-100 MG
Precision wipes	Kimtech Science, Kimberly-Clark Professional	Cat# 7552

**Deposited data**		

Mass spectrometry data	This paper	MassIVE: MSV000098910 https://doi.org/10.25345/C55Q4S029

**Software and algorithms**		

Xcalibur (ver.4.6.67)	Thermo Fisher Scientific	https://www.thermofisher.com/order/catalog/product/OPTON-30965
MSConvert (ver. 3.0.22354-a648f68)	ProteoWizard	Chambers et al.^49^
MZmine (ver. 4.0.8)	MZmine	https://mzio.io Schmid et al.^50^
SIRIUS (ver. 4)	Sirius	https://bio.informatik.uni-jena.de/software/sirius/
R (ver. 4.3.2)	R studio	http://www.rstudio.com/
qmtool package	R (ver. 4.3.2)	Joo and Himes^51^
sva package	R (ver. 4.3.2)	Leek et al.^52^
limma package	R (ver. 4.3.2)	Ritchie et al.^53^
mixOmics package	R (ver. 4.3.2)	Rohart et al.^54^

### Experimental model and study participant details

The EDDY program was carried out in three elementary Schools in Vienna with pupils between 7–10 years of age, including an intervention School and two control Schools. This study focusses on the children in the intervention School, where the dual intervention program based on physical activity and nutritional education was performed. Parents provided written consent for their child’s participation in this study. Moreover, the children gave oral assent for their participation. Participation was voluntary, and no compensation was provided. The ability to drop out was allowed at any time. A total of 54 children participated in this study, being a non-representative sample of 3^rd^ and 4^th^ grade pupils in Vienna.^15^^,^^18^ Details about the study cohort and the parents nationalities (Austrian or non-Austrian) are provided in Table S1. Ethical approval was obtained from the Ethical Committee of the Sigmund Freud University, Vienna, Austria (PAFGRW9O@EFQV885378, 15 Nov 2016). We obtained complete data sets from 31 children, representing a 57% response rate. Of those, 23 children presented with normal weight (9 female, 14 male) and 8 with overweight, including obesity (3 female, 5 male, Table 1). Data on height (cm) and bodyweight (kg) of volunteers were collected in June 2022, as well as 2024, and was measured using a TANITA scale, and a stadiometer (SECA 213, Germany). Later, BMI was calculated as kg/m^2^ (Table 1). The CDC 2000 growth chart for 2 to 20 years, stratified by sex and age, was used to classify the cohort.^55^ Specifically, children scoring a BMI above the 90^th^ percentile for their respective sex and age bracket were labelled as overweight. In this study, the term overweight also includes the obesity category. Hormonal differences between the sexes at pre-puberty age were considered negligible.

#### EDDY intervention

The EDDY program focuses on young children (mainly 7–10 years), as preventive effects have proven favourable in this age group.^56^ The original 6-month dual intervention program of the EDDY study^14^ was extended to 2×6 months in the School years 2022/2023 and 2023/2024. In Austria, one school year lasts from the beginning of September to the end of June. The nutrition intervention consisted of 10 sessions per School year and focused on age-appropriate nutrition education and its practical application in everyday life. It covered different topics through theoretical lessons and practical activities such as cooking and food tastings. Teachers were involved in the planning process and newsletters were sent to parents to reinforce what had been taught and to integrate healthy eating habits into family life. The physical activity intervention combined practical sports experience with theoretical education in 10 sessions per School year (2×45 min lessons every 2 weeks). It included strength and endurance training. Additionally, a newsletter was also sent to parents. In the School year 2022/2023, the intervention started in December 2022 and in the School year 2023/2024, the intervention started on October 2023. The intervention was completed in June 2024.^14^^,^^15^^,^^18^

### Method details

#### Eccrine sweat collection

Eccrine sweat samples were collected over the 2-year intervention in the intervention School in December 2022 (t1, baseline), May 2023 (t2) and May 2024 (t3). We collected 134 eccrine sweat samples of 54 children and obtained complete sample sets of 31 children (Table 1). Circular sampling units, punched with a 0.5 in. diameter from precision wipes (Kimtech Science, Kimberly-Clark Professional, USA), were pre-wetted with aqueous solution (3 μL, LC-MS grade H_2_O) and stored in labelled Eppendorf tubes at room temperature until use. Eccrine sweat samples were collected as follows: The hands were rinsed with warm tap water and subsequent dried using disposable paper towels. After a lag time of 1 min, the sampling unit was firmly held for 1 min between thumb and index+middle fingers. Then, the sampling unit was transferred back to the Eppendorf tube and stored at 4 °C until further processing.

#### Eccrine sweat metabolomics

##### Sample processing

Aqueous solution (120 μL, VWR Chemicals, LC-MS grade, Rosny-sous-Bois FR) containing caffeine-d9 (1 pg⋅μL^-1^) and 0.2% formic acid (FA, VWR Chemicals, LC-MS grade, Leuven BE) was added to the sampling unit in the Eppendorf tube. Metabolites were extracted for 30 s in an ultrasonic bath (100%, 25 °C, SONOREX DIGITAL 10P, BANDELIN electronic GmbH & Co. KG). The supernatant was transferred into HPLC vials equipped with a 200 μL V-shape glass insert (both Macherey-Nagel GmbH & Co.KG) to be analyzed by LC-MS/MS. Process blanks that represent blank extractions of the sampling unit were carried out and analysed as well.

##### Data acquisition

A 1290 Infinity II UHPLC System (Agilent) was coupled to an Exploris 480 mass spectrometer (Thermo Fisher Scientific) by an Optamax NG H-ESI (Thermo Fisher Scientific). A Kinetex XB-C18 column (100 Å, 1.7 μm, 100 × 2.1 mm, Phenomenex Inc.) was used for chromatographic separation. Mobile phase A consisted of water (VWR Chemicals, LC-MS grade, Rosny-sous-Bois FR) with 0.2% FA (VWR Chemicals, LC-MS grade, Leuven BE) and mobile phase B of methanol (MeOH, Honeywell, LC-MS grade, Seelze DE) with 0.2% FA. The following gradient was employed: 3% B for 0.15 min, 3–5% B from 0.15–0.30 min and 5–40% B from 0.30–4.50 min, followed by a column washing phase of 1.4 min at 80% B and a re-equilibration phase of 1.6 min at 3% B, resulting in a total runtime of 7.5 min. Flow rate was set to 500 μL⋅min^-1^, the column temperature to 40 °C and the injection volume was 10 μL. Electrospray ionization was performed in positive and negative ionization mode with a resolution of 60’000 FWHM at m/z 200, automatic injection time and an AGC target of 1e6. Selection for HCD fragmentation applying 50% normalized collision energy was done with a data dependent top 2 approach. Fragments were analyzed at a resolution of 30’000 FWHM at m/z 200, automatic injection time and an AGC target of 1e5. An extensive exclusion list from background ions and dynamic exclusion for 6 s minimized redundant MS2 scans. The instrument was controlled using Xcalibur software (ver. 4.6.67, Thermo Fisher Scientific).

### Quantification and statistical analysis

#### Raw data processing

Acquired raw data was converted to the mzML file format using MSConvert (ver. 3.0.22354-a648f68) from the ProteoWizard toolkit.^49^ Converted files were processed through MZmine (ver. 4.0.8) to receive a retention time aligned feature table with area values across all samples.^50^ MS2 precursor/fragment data was also exported from MZmine to be analyzed in SIRIUS (ver. 4) for the purpose of feature identification.^57^ Quantitative and qualitative data was combined in R (ver. 4.3.2) and further processed using the qmtools package.^51^ Features exceeding a value of 20% missing values in both testing conditions (normal weight vs overweight) were cut from the data set. Samples were normalized by tyrosine signal, followed by probabilistic quotient normalization (PQN). Missing values were imputed using a left-censored gaussian distribution approach, with values assigned from the 0.01 quantile to account for low-abundant features. Finally, batch correction was applied with the sva package’s improved ComBat function utilizing an empirical Bayes method to align data between timepoints.^52^ Additionally, redundant entries due to different ionic species of the same molecule were filtered for the most abundant feature using the annotations provided by SIRIUS. In total, 45’183 features were detected in the dataset of which 20’782 metabolites were putatively identified using the *de novo* search in SIRIUS. A categorical filter removed features detected in ˂80% of cases in at least one group (overweight or normal weight) and an additional filter step removed duplicate annotations from in-source fragmentation events, yielding 5’952 features. The detected metabolites from the process blanks were subtracted from the sample data. A 3-fold hard cut-off between process blank intensity and sample intensity was employed. Finally, metabolites with a confidence score ˂0.6 were removed, resulting in the 284 annotated metabolites with robust coverage and high confidence of identification.

#### Description of statistical analysis

The description of the study cohort is given as arithmetic means with standard deviations using the given number of participants in each group (Table 1): Total cohort (n = 54), total with complete sample sets (n = 31) and of those female (n = 12) and male (n = 19). A total of 284 metabolites were annotated in 134 acquired sweat metabolomes of 54 children. Differential analysis was performed using the mixOmics and limma packages in R.^53^^,^^54^ The 284 metabolites were used for partial least-squares discriminant analysis (PLS-DA) of all available participants at baseline (t1) according to normal weight (NW, n = 37, blue) and overweight (OW, n = 10, red) children (legend Figure 1B). Variable importance in projection scores (VIPs) were extracted from the PLS-DA model. Selected molecules among the most important predictors with an identification confidence of >60% were then further monitored across all timepoints. Statistical significance of BMI changes for NW (n = 22) and OW (n = 7) children were calculated using an unpaired t-test with Welch’s correction (legend Figure 1C). Statistical significance of abundance differences of metabolites in NW (n = 23) and OW (n = 8) children was calculated by fitting linear models using the limma package in R (legend Figures 2A and 3). Correlations of cyclamate abundance in eccrine sweat with BMI for OW children are shown for baseline (t1, n = 8) and after completion of the intervention (t3, n = 6, legend Figure 2B). Statistical tests in the figures are represented by an asterisk (∗) and generally refer to a p-value < 0.05.

## Supplemental information

Table S1. Data associated with cohort, PLS-DA (related to Figure 1), metabolomics data, and LIMMA result
